# Estimating the causal impact of chewing disability on depressive symptoms mediated by loneliness: a longitudinal marginal structural model study of older adults in Singapore

**DOI:** 10.1093/geroni/igaf100

**Published:** 2025-09-16

**Authors:** John Rong Hao Tay, Gustavo G Nascimento, Angelique Chan, Rahul Malhotra, Maurizio S Tonetti, Marco A Peres

**Affiliations:** Health Services Research and Population Health Programme, Duke-NUS Medical School, Singapore, Singapore; Department of Restorative Dentistry, National Dental Centre Singapore, Singapore, Singapore; National Dental Research Institute Singapore, National Dental Centre Singapore, Singapore, Singapore; Oral Health Academic Clinical Programme, Duke-NUS Medical School, Singapore, Singapore; Health Services Research and Population Health Programme, Duke-NUS Medical School, Singapore, Singapore; Centre for Ageing Research and Education, Duke-NUS Medical School, Singapore, Singapore; Health Services Research and Population Health Programme, Duke-NUS Medical School, Singapore, Singapore; Centre for Ageing Research and Education, Duke-NUS Medical School, Singapore, Singapore; Shanghai Perio-Implant Innovation Center, Institute for Oral Craniofacial and Sensory Research, Shanghai Key Laboratory of Stomatology—National Clinical Research Center for Oral Diseases, Shanghai Ninth People’s Hospital, Shanghai Jiao Tong University School of Medicine, Shanghai, China; European Research Group on Periodontology, Genoa, Italy; Health Services Research and Population Health Programme, Duke-NUS Medical School, Singapore, Singapore; National Dental Research Institute Singapore, National Dental Centre Singapore, Singapore, Singapore

**Keywords:** Epidemiology, Oral health, Mastication, Depression

## Abstract

**Background and Objectives:**

Chewing disability may contribute to depressive symptoms in older adults, but causal pathways, accounting for time-varying confounding factors, remain unexplored. Previous research shows an association between chewing disability, loneliness, and depression. This study examines the causal relationship between chewing disability and clinically significant depressive symptoms (CSDS) and whether loneliness mediates this association among older adults.

**Research Design and Methods:**

In total, 1,277 participants aged ≥60 years, without CSDS at baseline, were selected from a nationally representative study of older adults in Singapore (2009–2015, three waves). Marginal structural models were utilized to estimate total and indirect effects of chewing disability and CSDS over time, where loneliness was treated as a mediator.

**Results:**

Across the study period, 10.3% developed CSDS, 40.7% experienced loneliness, and 33.6% had chewing disability. Individuals with chewing disability had a 48% higher risk of CSDS (RR: 1.48, 95% CI: 1.15–1.82), and the indirect effect through loneliness was 26% (RR: 1.26, 95% CI: 0.99–1.53; 17.3% of the total effect). Nonetheless, the estimates varied by the operationalization of chewing disability and loneliness. A broader definition of chewing disability showed stronger total effects (RR: 1.57, 95% CI: 1.24–1.91), while a stricter loneliness threshold had a greater indirect effect (RR: 1.70, 95% CI: 1.30–2.09; 21.8% of the total effect).

**Discussion and Implications:**

Chewing disability increases the risk of CSDS among older adults, with partial mediation by loneliness. Further research on oral rehabilitative interventions that improve chewing function and mitigate depressive symptoms in older adults is needed.

Innovation and Translational Significance Chewing disability is a common but under-recognized risk factor for depressive symptoms in aging populations. This study demonstrates that chewing disability increases the risk of clinically significant depressive symptoms in older adults, with loneliness acting as a partial mediator. By modeling these relationships longitudinally, this approach moves beyond conventional regression to provide a more rigorous causal framework. Translationally, it highlights a psychosocial pathway linking oral function to mental health and underscores the importance of integrating oral health with geriatric mental health care. At a population level, policies that promote oral health equity may also improve psychological well-being in aging societies.

## Background and objectives

Chewing disability is broadly defined as the reduced ability to fragment food efficiently and safely (Gonçalves et al., 2021). Tooth loss remains its most direct and well-established determinant, especially when molars are missing, as this reduces bite force and compromises chewing efficiency (Lahoud et al., 2023). However, non-dental factors, such as sarcopenia, salivary hypofunction, and frailty, have been implicated as well (Furuhashi et al., 2024). In addition, socioeconomic disadvantage, low dental utilization, and poor nutritional intake are associated with higher rates of chewing disability (Lahoud et al., 2023). Prevalence estimates suggest that around 35% of older adults experience chewing disability, both in community settings and long-term care facilities (Abreu et al., 2023; Franco et al., 2025).

Chewing disability is hypothesized to affect depression through several pathways. Nutritionally, it can lead to a reliance on softer, processed foods that are often nutrient-poor, resulting in deficiencies in key vitamins and minerals involved in mood regulation (Lang et al., 2015). This nutritional imbalance may contribute to depression by disrupting pathways involved in inflammation, oxidative stress, and the gut-brain axis (Marx et al., 2021). Oral inflammation may also trigger a hyperinflammatory systemic response and altered cortisol secretion via hypothalamic-pituitary-adrenal axis dysfunction (Marx et al., 2021).

Beyond nutritional and inflammatory mechanisms, psychosocial pathways may significantly contribute to this association. Pain during mastication can lead to a reduced sense of well-­being, contributing to depressive symptoms. Difficulty eating in social settings can lead to embarrassment and anxiety, reinforcing a sense of functional impairment (Rouxel et al., 2017). Difficulty eating may also lead to avoidance of social engagements, which increases the probability of becoming lonely.

Loneliness is a distressing emotional state that arises from a discrepancy between an individual’s desired and actual social relationships (Mann et al., 2017). Several hypothesized mechanisms have been proposed to explain its link with depression. Psychologically, loneliness may induce negative cognitive biases, including a heightened attention to negative social interactions, past adverse social interactions, and negative self-­evaluations (Cacioppo et al., 2003, 2006; Lee et al., 2021). Loneliness has been identified as a risk factor for the development of depression in older adults, and given the high prevalence of both conditions, reducing loneliness may play an important role in improving mental health outcomes in older adults (Lee et al., 2021).

Loneliness is common among individuals with functional impairments due to reduced autonomy and barriers to social interaction (Gómez-Zúñiga et al., 2022). Functional difficulties, including those related to oral health, are associated with poorer mental well-being, with loneliness serving as a key mediator (Barman et al., 2024). This is supported by findings that poorer diet quality, which is closely dependent on chewing ability, is consistently associated with loneliness (Hanna et al., 2023). Therefore, loneliness may not only co-occur with chewing disability and depression, but may also mediate their association.

Few studies have explored loneliness as a mediator between chewing disability and depression, despite eating being a fundamental social activity. In a collectivist Asian society like Singapore, communal dining fosters meaningful social engagement and personal identities (Plastow et al., 2015). Singapore’s hawker culture, an integral part of daily life where individuals from all backgrounds gather in open-air complexes housing dozens of foods stalls where diverse and affordable local foods are prepared and shared in communal spaces, has been recognized by UNESCO as an intangible cultural heritage. This culture is embedded in the identity of Singaporeans and exemplifies a deep-rooted tradition of shared meals as a social ­cornerstone. For individuals with chewing disability, difficulties in participating in these social experiences may heighten loneliness and contribute to depression. Examining the role of chewing ability may offer socially integrated approaches to improve the mental health of older adults with impaired oral function.

In Singapore, dental care is delivered through a mixed private–public system, with the private sector accounting for approximately 80% of dental visits. While there is no universal coverage, subsidies such as the Community Health Assist Scheme provide means-tested subsidies for basic dental services for participating clinics, while age-specific subsidies provide additional subsidies for older people (Tada et al., 2024). Those with complex treatment needs are referred to one of the two national specialty dental centers, where they still receive subsidized care. These policies have led to improved uptake of dental services among older adults, but challenges remain. Although the 2019 National Adult Oral Health Survey in Singapore did not assess chewing disability directly, it reported that the prevalence of edentulism among older adults has remained stable at 12.5% since 2003, with only 35.7% of older adults found to have a functional dentition (retention of ≥21 natural teeth) (Sim et al., 2024).

Between 1990 and 2019, the number of older adults (≥60 years old) with depressive disorders increased by 116%, reaching 58.9 million globally (Rong et al., 2024). The rising burden of mental health conditions is paralleled by increasing oral functional impairments in later life (Aida et al., 2022). Given these interconnections, it is important to examine how chewing disability, loneliness, and depression interact with one another in older adults.

However, most studies rely on cross-sectional designs, limiting causal inference. Establishing causality may inform interventions on preserving chewing function to mitigate depression, especially in older adults. Additionally, the potential for cumulative effects and time-varying confounders remains underexplored. Cumulative effects refer to exposures that build up over time, while time-varying confounders, such as changes in psychosocial and physical health, can influence both the exposure and outcome at different time points, potentially resulting in bias in associations if not properly accounted for. This study investigates the causal effect of chewing disability on the development of clinically significant depressive symptoms (CSDS; defined later) among older adults and assesses whether loneliness mediates this relationship.

## Research design and methods

The article adhered to the Strengthening the Reporting of Observational Studies in Epidemiology (STROBE) guidelines.

### Data and analytical sample

**Figure 1.**
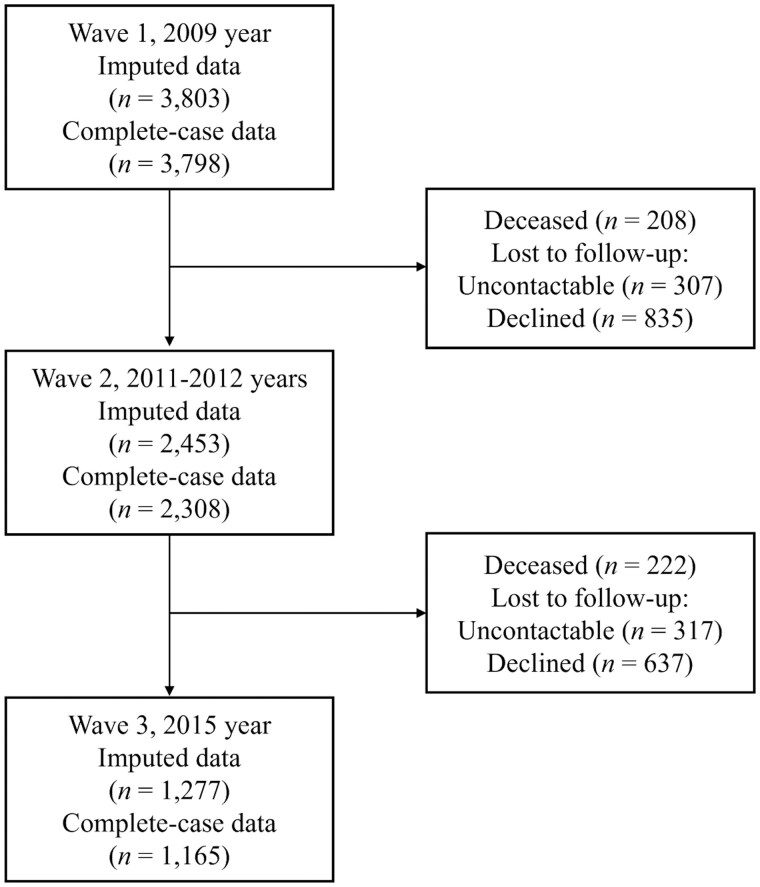
Flowchart of the three waves of the PHASE from baseline (2009) to the end of follow-up (2015).

This study drew upon data from all three waves of the Panel on Health and Ageing among Singaporean Elderly (PHASE), a longitudinal, nationally representative study of community-­dwelling Singapore citizens and permanent residents aged 60 years and older (Chan et al., 2019). The baseline was conducted in 2009, with subsequent follow-ups in 2011–2012 and 2015 (Figure 1). Participants with CSDS at baseline were excluded. Participants with no missing data on the outcome and exposure from all three waves were included.

### Exposure

Chewing disability was evaluated as a time-varying exposure variable across Waves 1 and 2. Participants were asked, “The following foods are ordered from hardest to softest to chew. What is the hardest group you are able to bite and chew?” Response options were structured based on six ordinal food groupings reflecting the relationship between food toughness and the muscle activity required for mastication. The classification was adapted to the local context by evaluating the toughness of commonly consumed foods among older adults in Singapore using a texturometer (Nascimento et al., 2024). Details on the food groupings are found in Supplementary Table 1 (see online supplementary material).

For analysis, chewing ability was dichotomized: participants who reported being able to chew the toughest food group were classified as having no chewing disability, while those who reported difficulty with any of the remaining food groups were categorized as experiencing chewing disability (Nascimento et al., 2024).

### Mediator

Loneliness was assessed as a time-varying variable across Waves 1 and 2 using an adapted Three-Item Loneliness Scale (TILS) (Hughes et al., 2004). Participants were asked about their experiences of social connection through three questions: how frequently they felt a lack of companionship, how often they felt excluded, and how often they felt isolated from others. Each question was scored on a scale from 0 (never) to 4 (always), with higher values indicating greater loneliness. This variable was dichotomized, with participants with any score of one and above classified as having some measure of loneliness (Chan et al., 2015).

### Confounders

Time-invariant confounders included demographic variables for sex (male/female), ethnicity (Chinese/Malay/Indian/Others), education (primary school or below/above primary school), and housing type (1–2 room government-built housing/3-room government-built housing/4–5 room government-built housing or private housing). Time-varying confounders included age (in years), mobility difficulty, cerebrovascular disease, coronary heart disease, diabetes mellitus, cancer, and social support network scores. Mobility difficulty (yes/no) was assessed using Nagi’s index of physical performance, which measures upper and lower limb function (Nagi, 1976). Participants were classified as having mobility difficulty if they reported at least one mobility limitation. Medical conditions were self-reported (yes/no), based on the question: “Have you been diagnosed by a medical professional with [condition]?” Social support network scores were assessed using a modified version of the Lubben Social Network Scale-Revised, ranging from 0 to 60, with higher scores indicating greater perceived social support from family members outside the household and friends (Chan et al., 2018; Lubben et al., 2006).

### Outcome

The outcome was CSDS at Wave 3. CSDS was evaluated using the 11-item version of the Center for Epidemiologic Studies—Depression (CES-D) Scale, which captured the participants’ emotional and psychological states over the past week. The scale comprised 11 items: “My appetite was poor”; “I felt depressed”; “I felt that everything I did was an effort”; “My sleep was restless”; “I felt happy”; “I felt lonely”; “I felt people were unfriendly”; “I enjoyed life”; “I felt sad”; “I felt that people disliked me”; and “I could not get ‘going’” (Kohout et al., 1993). Each item was rated on a three-point scale, where 0 indicated none or rarely, 1 indicated sometimes, and 2 indicated often. The total CES-D score ranged from 0 to 22, with higher scores reflecting a greater extent of depressive symptoms. For the primary analysis, the outcome was dichotomized, with a score of 7 or higher indicating being positive for clinically significant depressive symptoms (Malhotra et al., 2011; Yokoyama et al., 2010).

### Statistical analysis

**Figure 2.**
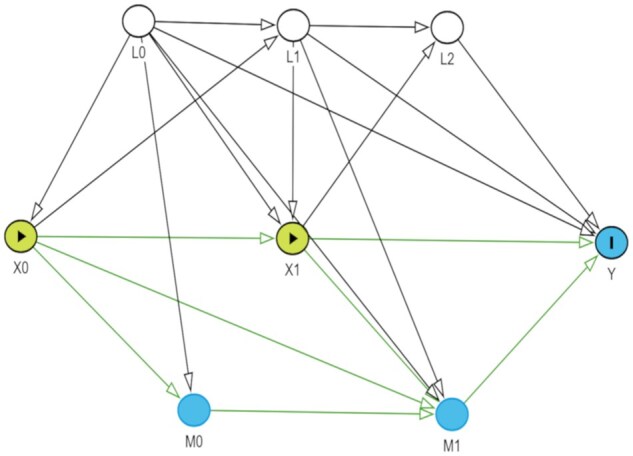
Proposed longitudinal mediation analysis framework. L0, L1, L2 (Time-invariant confounders): sex, ethnicity, education, housing type; L0, L1, L2 (Time-varying confounders): mobility difficulty, cerebrovascular disease, coronary heart disease, diabetes mellitus, cancer, social support network; X0, X1 (Exposures): chewing disability at Wave 1 (2009) and Wave 2 (2011–2012); M0, M1 (Mediators): loneliness at Waves 1,2; Y (Outcome): clinically significant depressive symptoms (CSDS) at Wave 3. Green arrows indicate causal paths.

Analyses were based on a longitudinal mediation analysis framework to determine causal pathways between chewing disability and CSDS, with loneliness as the mediator (Figure 2).

To account for time-varying confounding, marginal structural modeling (MSM) was utilized via inverse probability treatment weighting (IPW). IPW assigns weights to individuals based on their probability of receiving the exposure, being censored, and experiencing the mediator at each time point, creating a pseudo-population, where the exposure is independent of these time-dependent confounders (Robins et al., 2000). The weighted dataset is then used to estimate potential outcomes and causal mediation effects using weighted regression models. To adjust for these confounders, the following weights were computed (Robins et al., 2000):


$$\mathrm{Exposure}\;\mathrm{weight}\;(\mathrm{Wave}\;1\;\mathrm{and}\;2):\;{SW}_{t}^{X}=\frac{P(X_{t}\;|\;X_{t-1})}{P(X_{t}\;|\;X_{t-1},\;L_{t})\;}$$

$$\mathrm{Mediator}\;\mathrm{weight}\;(\mathrm{Wave}\;1\;\mathrm{and}\;2):\;{SW}_{t}^{M}=\frac{P(M_{t}\;|\;{X_{t},\;M}_{t-1})}{P(M_{t}\;|\;{X_{t},\;M}_{t-1},\;L_{t})\;}$$

$$\mathrm{Censoring}\;\mathrm{weight}\;(\mathrm{up}\;\mathrm{to}\;\mathrm{Wave}\;3):\;{SW}_{t}^{C}=\frac{P(C_{t}\;|\;C_{t-1})}{P(C_{t}\;|\;C_{t-1},\;L_{t})\;}$$

These weights were then cumulatively combined across all time points to account for time-varying confounding in the model. The final stabilized weight across the three time points were computed as:


$$SW_{\text{total}}=(\prod_{t=1}^{2}SW_{t}^{X})\times (\prod_{t=1}^{2}SW_{t}^{M})\times (\prod_{t=2}^{3}SW_{t}^{C})$$

Weighted pooled Poisson regression with a log link and robust standard errors (Zou, 2004) was then used to estimate the total and indirect total effects of chewing disability on CSDS and was expressed as follows:


$$\mathrm{Total}\;\mathrm{effect}\;\mathrm{model}:\;\log\;P\;(Y\;=\;1|\;\bar{X}\;=\;\bar{x})\;=\;\beta_{0}\;+\;\beta_{1cum}(\bar{x})$$

$$\mathrm{Direct}\;\mathrm{effect}\;\mathrm{model}:\;\log\;P\;(Y\;=\;1|\;\bar{X}\;=\;\bar{x})\;=\;\beta_{0}\;+\;\beta_{1cum}(\bar{x})+\;\beta_{2cum}(\bar{m})$$

This can be interpreted as the population-averaged (marginal) mean of the counterfactuals when the population is exposed, allowing a causal interpretation by assuming consistency, exchangeability, and positivity (VanderWeele, 2009). The coefficients $\beta_{0}\;$ and $\;\beta_{1cum}$ represent the log risk, with $\beta_{1cum}$ exponentiated to provide the relative risk. The proportion mediated by loneliness was determined by comparing the coefficient of the indirect effect with the total effect.

Missing data were imputed using chained equations to produce 10 imputed datasets (Van Buuren & Groothuis-Oudshoorn, 2011). Participants with missing baseline values for chewing exposure and CSDS status were excluded prior to imputation. Imputation was applied to participants who remained uncensored through Wave 3, under the missing at random assumption. Results from the imputed datasets were pooled using Rubin’s rules (Van Buuren & Groothuis-­Oudshoorn, 2011). For complete case analyses, precision of the observed estimates was quantified using bias-corrected and accelerated bootstrapping with 5,000 replicates to compute 95% confidence intervals (Chernick & LaBudde, 2014). Weight distributions were assessed both before and after truncation by examining the mean, standard deviation, and extreme percentiles (1st and 99th) of the stabilized weights. Density plots were used to visualize weight distribution and the presence of outliers. The positivity assumption was assessed by examining the distribution of predicted probabilities from the exposure and mediator models at Waves 1 and 2, with a lack of extreme predicted probabilities (<5% or >95%) suggesting that participants had a non-negligible probability of receiving each exposure level across covariates (Cole & Hernán, 2008).

For sensitivity analyses, E-values were calculated to assess the impact of unmeasured confounding (VanderWeele & Ding, 2017). Chewing disability was also operationalized differently, with Groups 1 and 2 classified as having no chewing disability, based on the ordinal food toughness scale described earlier. Loneliness was alternatively operationalized using a stricter threshold, with a TILS score of 4 or higher classified as “mostly lonely” to minimize misclassification (Chan et al., 2015). To explore potential bias from differential dropout, a sensitivity analysis excluded participants with baseline cognitive impairment, defined as a score of 3 or higher on the 10-item Short Portable Mental Status Questionnaire, adjusted for interviewer effects (Malhotra et al., 2015). This analysis aimed to determine whether dropout influenced the observed associations, particularly if individuals with chewing disability were more likely to leave the study due to cognitive decline. In addition, to account for loss to follow-up and to assess robustness under a missing-not-at-random assumption, sensitivity analysis was done by imputing the CSDS outcome for censored individuals under two extreme scenarios: (a) a best-case scenario assuming none of the dropouts developed CSDS and (b) a worst-case scenario assuming all of the dropouts developed CSDS. MSMs were then re-estimated using the same stabilized IPWs to examine the total, direct, and mediated effects.

The outcome and mediator were also analyzed as continuous variables within the MSM framework, using CES-D and TILS scores, respectively. The same IPWs were used to fit the linear regression models with an identity link and robust standard errors. In this sensitivity analysis, no participants were excluded based on baseline CES-D scores, allowing inclusion of those with early-onset or preexisting CSDS to enhance the robustness of findings. Mean differences were converted to approximate relative risks to facilitate interpretability, and E-values were calculated to assess robustness to unmeasured confounding (VanderWeele & Ding, 2017).

All analyses were prepared and analyzed using R (version 4.4.2, R Core Team, Vienna, Austria), using the *survey* package for MSM estimation and *boot* for bootstrapped confidence intervals.

## Results

At baseline, 734 participants (16.2%) with CSDS were excluded. There were a total of 3,803 and 2,453 participants at Wave 1 and Wave 2, respectively (Figure 1). The final sample for analysis consisted of 1,277 participants who were surveyed across all three waves (Table 1). Supplementary Tables 2–4 (see online supplementary material) report characteristics of participants using complete case analysis and distribution of missing data. Supplementary Table 5 (see online supplementary material) compares characteristics across Waves 1 to 3 using continuous CSDS and loneliness scores in the imputed and complete case analysis. The mean age at baseline was 70.4 (7.1) years. The prevalence of participants with CSDS at Wave 2 was 5.8%, increasing to 10.3% at Wave 3. Across Waves 2 and 3, higher rates of CSDS were observed among women with primary school or below, living in smaller government-built housing, experiencing mobility difficulty, and those reporting loneliness.

**Table 1.** Baseline and follow-up characteristics of participants with complete follow-up from Wave 1 (2009) through Wave 3 (2015), with missing data imputed using multiple imputation.

Characteristics	Wave 1 (2009)	Wave 2 (2011–2012) Wave 3 (2015)				
	No CSDS	No CSDS	With CSDS	No CSDS		With CSDS
**Age, mean (*SD*)**	70.4 (7.1)	72.2 (7.0)	73.3 (7.4)	76.3 (7.1)		77.0 (7.0)
**Sex, %**						
** Male**	46.5	47.2	35.7	47.2		40.4
** Female**	53.5	52.8	64.3	52.8		59.6
**Ethnicity, %**						
** Chinese**	76.0	75.9	78.7	76.2		75.1
** Malay**	15.3	15.6	9.9	15.2		15.8
** Indian**	7.7	7.4	11.4	7.6		7.6
** Others**	1.0	1.1	0.0	1.0		1.4
**Education, %**						
** Primary school or below**	67.6	67.2	72.0	66.5		75.6
** Above primary school**	32.4	32.8	28.0	33.5		24.4
**Housing, %**						
** 1–2 room government-built**	6.7	6.1	16.8	6.1		12.2
** 3 room government-built**	27.1	27.0	28.9	26.8		30.5
** 4–5 room government-built/private housing**	66.2	66.9	54.3	67.2		57.4
**Smoking, %**						
** Non-/ex-smoker**	89.5	90.9	91.6	92.0		95.3
** Current**	10.5	9.1	8.4	8.0		4.7
**Cerebrovascular disease, %**						
** Yes**	2.1	2.0	5.5	3.5		8.3
** No**	97.9	98.0	94.5	96.5		91.7
**Coronary heart disease, %**						
** Yes**	5.5	4.1	4.1	7.2		15.3
** No**	94.5	95.9	95.9	92.8		84.7
**Diabetes mellitus, %**						
** Yes**	21.7	22.6	34.0	28.7		36.1
** No**	78.3	77.4	66.0	77.3		63.9
**Cancer, %**						
** Yes**	1.9	0.9	2.5	3.8		3.5
** No**	98.1	99.1	97.5	96.2		96.5
**Mobility difficulty, %**						
** Yes**	29.5	31.6	51.0	51.0		72.7
** No**	70.5	68.4	49.0	49.0		27.3
**Social support network (modified LSNS-R), mean (*SD*)**	30.2 (12.6)	29 (10.6)	23.9 (9.4)	27.1 (11.5)		23.8 (10.5)
**Chewing disability, %**						
** Yes**	16.8	25.3	49.4	32.4		43.6
** No**	83.2	74.7	50.6	67.6		56.4
**Loneliness (TILS ≥1), %**						
** Yes**	41.7	35.5	73.1	36.8		74.4
** No**	58.3	64.5	26.9	63.2		25.6

*Note.* CSDS = clinically significant depressive symptoms; LSNS-R = Lubben Social Network Scale-Revised; *N* = number; *SD* = standard deviation; TILS = Three-Item Loneliness Scale.

The proportion of individuals with both chewing disability and CSDS was 49.4% and 43.6% at Waves 2 and 3, respectively. Among those with CSDS, the prevalence of loneliness was 73.1% at Wave 2 and 74.4% at Wave 3.

Applying marginal structural modeling on the imputed data, the total effect of chewing disability corresponded to a 48% increased risk of CSDS at the end of follow-up (RR: 1.48, 95% CI: 1.15–1.82) (Table 2). When adjusted for loneliness as a mediator, the direct effect of chewing disability corresponded to a 26% increased risk of CSDS (RR: 1.26, 95% CI: 0.99–1.53), although the confidence interval includes the null, indicating some uncertainty in the estimate. Loneliness mediated 17.30% of the total effect in the imputed data. The E-values indicated robustness to unmeasured confounding, suggesting that an unmeasured confounder with a risk ratio of 2.33 with both exposure and outcome was needed to explain away the total effect, while E-values of 2.15 and 1.82 were needed to nullify the direct and indirect effects, respectively. In the complete case analysis, the total effect of chewing disability on CSDS was similar, corresponding to a 42% increased risk (RR: 1.42, 95% CI: 1.10–1.81). The direct effect was similar at 39% (RR: 1.39, 95% CI: 1.07–1.76), while mediation accounted for 8.22% of the total effect (Table 2). Summary diagnostics showed that weight truncation effectively reduced extreme weights, resulting in a more stable distribution (Supplementary Table 6 and Supplementary Figure 1, see online supplementary material). Predicted probabilities from the exposure and mediator models at Waves 1 and 2 suggested that the positivity assumption was generally satisfied. While all mediator weights fell within the central range, the exposure weights were slightly right-skewed, and the exposure model at Wave 1 yielded a small proportion (1.98%) of predicted probabilities below 0.05 (Supplementary Figure 2, see online supplementary material).

**Table 2.** Estimated impact of chewing disability on CSDS using inverse probability treatment weighting and marginal structural models.

Variable	Imputed data (*n *= 1,277)				Complete case analysis (*n *= 1,165)			
	RR	95% CI	E-value	Proportion contributed	RR	95% CI	E-value	Proportion contributed
Total cumulative effect (Model 1)	1.48	1.15–1.82	2.33	100%	1.42	1.10–1.81	2.20	100%
Direct cumulative effect (Model 2)	1.40	1.04–1.76	2.15	82.70%	1.39	1.07–1.76	2.12	91.78%
Cumulative mediator effect (loneliness)	1.26	0.99–1.53	1.82	17.30%	1.22	0.94–1.58	1.73	8.22%

*Note.* Models adjusted for age, sex, ethnicity, education, housing type, cerebrovascular disease, coronary heart disease, diabetes mellitus, cancer, and social support network. Model 2 additionally adjusts for loneliness as a mediator. CIs for the complete-case analysis were obtained using nonparametric bootstrap with 5,000 replicates. CSDS = clinically significant depressive symptoms; CI = confidence interval; RR = relative risk.

Sensitivity analyses using the alternative operationalization of chewing disability (Groups 1 & 2 = no disability) yielded consistent findings (Supplementary Table 7, see online supplementary material), with stronger effects observed. The total effect increased to a 57% higher risk of CSDS (RR: 1.57, 95% CI: 1.24–1.91), and the direct effect rose to 50% (RR: 1.50, 95% CI: 1.18–1.83). The mediated effect remained uncertain (RR: 1.22, 95% CI: 0.95–1.48), with loneliness accounting for 12.5% of the total effect. When loneliness was operationalized using a stricter threshold (TILS ≥4), the total effect of chewing disability remained consistent (RR: 1.44, 95% CI: 1.12–1.77). However, the independent effect of loneliness increased (RR: 1.70, 95% CI: 1.30–2.09), with mediation explaining a larger proportion of the total effect (21.79%) (Supplementary Table 8, see online supplementary material). Sensitivity analysis excluding participants with baseline cognitive impairment yielded similar estimates, suggesting that potential differential dropout due to cognitive decline among those with chewing disability did not substantially alter the observed associations (Supplementary Table 9, see online supplementary material). Sensitivity analyses addressing nonrandom loss to follow-up at Wave 3 showed that the total effect persisted under best-case (RR: 1.53, 95% CI: 1.19–1.97) and worst-case assumptions (RR: 1.13, 95% CI: 1.05–1.22). Full estimates for total, direct, and indirect effects are presented in Supplementary Table 10 (see online supplementary material). When CSDS and loneliness were re-analyzed as continuous variables using the imputed dataset, chewing disability was associated with a mean increase of 0.90 points in CES-D score (95% CI: 0.61–1.19), corresponding to an approximate RR of 1.29 (95% CI: 1.19–1.40) and an E-value of 1.90. Loneliness was associated with an increase of 0.17 points in CES-D score (95% CI: 0.09–0.24), corresponding to an approximate RR of 1.05 (95% CI: 1.03–1.07), mediating 26.5% of the total effect (Supplementary Table 11, see online supplementary material).

## Discussion and implications

This study demonstrated that chewing disability increases the risk of CSDS in older adults, through both total and direct pathways. However, the extent to which loneliness mediated this relationship varied across model specifications. When a more lenient definition of chewing disability was utilized, both the total and direct effect of chewing disability increased in magnitude, suggesting that milder impairments in chewing disability may still directly impact CSDS. However, the contribution of loneliness remained uncertain. When loneliness was operationalized using a stricter threshold, its indirect effect in the causal pathway was stronger. This may be because the model identified individuals with more severe loneliness, who are more likely to be affected by chewing disability and develop CSDS. Sensitivity analyses using continuous CES-D and TILS scores revealed a stronger mediating role for loneliness. Unlike the binary CSDS definition, the continuous approach retained individuals with subthreshold or early-onset depressive symptoms rather than excluding them, which appears to uncover mediation that dichotomization obscured. These findings show the potential mental health implications of chewing disability in older adults and suggest that preserving chewing function plays a role in reducing depressive symptoms.

Depression is a significant public health concern and is associated with increased healthcare utilization and poorer outcomes in comorbidities like cardiovascular disease and cancer (McLaughlin, 2011). The Global Burden of Disease 2019 study ranks depressive disorders as the leading cause of disability attributed to mental disorders (Global Burden of Disease Collaborative Network, 2022). Chewing disability may be an under-recognized factor in late-life depression. Addressing oral health issues, such as improved dental care access and oral rehabilitation, may mitigate this burden. These findings also suggest that oral health assessments may be beneficial among older adults presenting with depressive symptoms.

While oral health interventions may reduce CSDS risk, their economic justification remains complex. Prosthetic rehabilitation may be costly and inaccessible in lower-income populations (Vernazza et al., 2021). Preventive care such as subsidized dental maintenance may help reduce tooth loss and preserve chewing ability at a lower cost, though tradeoffs with other public health priorities must be considered (Vernazza et al., 2021).

These findings build on previous research associating chewing disability with loneliness (Maki et al., 2024; Rouxel et al., 2017) and CSDS (Ohi et al., 2022), but advance the literature by applying a longitudinal framework with causal modeling. While most prior studies were cross-sectional, this study used MSMs and IPW to estimate both total and direct effects, accounting for time-varying and exposure-mediator feedback. Standard regression methods assume all confounders are baseline covariates and cannot adequately handle scenarios where variables like loneliness may be mediated and influenced by prior exposure, introducing bias (Robins et al., 2000). Generalized estimating equations are commonly used in longitudinal studies to account for repeated measures within individuals, but they also do not adjust for confounding affected by prior exposure. Similarly, conventional mediation models assume that all confounders are measured at baseline and remain constant over time, relying on indirect effect estimation and not accounting for exposure-mediator feedback loops. Although loneliness did not consistently demonstrate a robust mediating effect across models, this analytic strategy nonetheless enabled a clearer characterization of the role of chewing disability in the development of CSDS.

MSM also accounts for censoring, reducing bias due to participant dropout, which is common in longitudinal studies (Hernán et al., 2004). Additionally, multiple imputation minimized bias from missing data. However, the substantial attrition of participants over the three waves raises concerns about potential nonrandom dropout that censoring weights may not fully account for. To address this, we assessed differential dropout by baseline cognitive status and found no evidence that participants with chewing disability were more likely to be lost to follow-up due to cognitive decline. Additionally, sensitivity analysis under missing-not-at-random assumptions showed that effect estimates were reduced under the worst-case scenario where all dropouts were assumed to have developed CSDS, but the overall direction of association remained significant, with confidence estimates excluding the null. Finally, secondary analysis by modeling CSDS and loneliness as continuous scores avoided information loss inherent to dichotomization and enabled the inclusion of a larger sample, as participants with baseline CSDS were not excluded. modeling the outcome and mediator as continuous scores showed modest increases in depressive symptom scores associated with chewing disability. Although the mean increase of CES-D scores likely falls below the minimally clinically important difference, at a population level, even this modest upward shift moves the overall proportion of individuals crossing the clinical threshold of CSDS, explaining the observed elevated risk (RR ≈ 1.29).

The study also benefited from a well-defined population design drawn from a nationally representative cohort of older adults in Singapore, and the use of validated tools for assessing key variables, including chewing disability, loneliness, and CSDS. While the follow-up of six years may appear relatively short, this duration is advantageous in capturing time-varying confounding. Longer follow-up periods may introduce additional unmeasured confounding and increased loss to follow-up. Furthermore, differential mortality may introduce selection bias, as individuals with chewing disability in this older cohort may face a competing risk of death before developing depression (Tay et al., 2025).

There are limitations in this study. First, chewing disability was self-reported, which may introduce response bias. Individuals may overestimate or underestimate their chewing function based on personal perceptions rather than actual impairment. While objective assessments exist (e.g., two-color gum tests), they are not easily applicable in population-based settings, and epidemiological studies often classify chewing disability using self-reported difficulty with tough foods (Gonçalves et al., 2021).

Second, the CES-D Scale is a screening tool. While widely used in epidemiological studies, it relies on self-reported symptoms, which may inflate depressive scores, particularly in individuals already concerned about their mental health (Moon et al., 2017). Additionally, the CES-D Scale includes a loneliness item, which conceptually overlaps with the loneliness variable using the TILS. This, however, does not invalidate the findings, as the CES-D loneliness item reflects a transient state within the cluster of depressive symptoms measured at the final wave, while TILS captures a more stable and multidimensional measure of perceived social isolation, reinforcing their distinct conceptual roles (Wolska & Creaven, 2023). The CES-D Scale also does not exclusively assess CSDS but also captures symptoms of anxiety, which, while closely related, are distinct clinical entities (Moon et al., 2017). Furthermore, the specificity of the CES-D may be lower in community-dwelling persons, leading to a higher rate of false positives (Park & Yu, 2021).

Third, this study approach relies on the positivity assumption, where every covariate combination has a non-zero probability for each exposure and mediator level. A small proportion of predicted probabilities in the exposure model fell below 5%, indicating minimal risk of positivity violations. To further improve model stability, stabilized weights were truncated at the 95th percentile to limit their influence. Finally, despite adjustments for a broad range of confounders, residual confounding cannot be fully ruled out (e.g., time-invariant factors such as major life events or personality traits). Nevertheless, the E-value analysis indicated that moderate unmeasured confounding would be needed to negate the findings.

The study relied on several assumptions to enable causal inference. To assess the robustness of the findings, several sensitivity analyses were done. While this strengthens the credibility of the results, it is acknowledged that the assumptions cannot be fully verified in an observational study and should be interpreted with appropriate caution. This study also cannot directly elucidate the psychosocial or biological mechanisms between chewing function and CSDS. A possible explanation is that poor oral function, which can impair eating, speaking, and social engagement, leads to diminished self-worth and increased social withdrawal (Benyamini et al., 2004). It may also act as a chronic stressor, particularly for individuals already experiencing loneliness, reinforcing emotional distress and altering stress perceptions (Cacioppo et al., 2003).

These findings highlight the potential role of oral health in geriatric care in Singapore. This may include improving healthcare accessibility to improve oral health equity, such as mobile dental services and expanding dental subsidies (Tada et al., 2024). While regular dental visits have increased in Singapore since 2014 following the introduction of age-specific financial subsidies for older adults (i.e., the Pioneer and Merdeka Generation schemes), utilization remains skewed toward individuals with higher educational attainment and among Chinese compared with Malay and Indian ethnic groups (Allen et al., 2024). Moreover, the prevalence of edentulism has remained unchanged over the years, suggesting that structural barriers such as socioeconomic status, low oral health literacy, and limited perceived need continue to hinder effective treatment uptake.

The findings of this study are not directly generalizable to other geographic settings. Constructs such as loneliness and depression are shaped by cultural norms and social environments. Nonetheless, the results offer valuable hypotheses for future research in other rapidly aging societies with similar demographic and oral health challenges. Finally, while this study applies causal inference methods to demonstrate the impact of chewing disability on mental well-being, evidence on dental interventions in reducing depression risk remains limited.

## Conclusions

Chewing disability contributes to depressive symptoms, with loneliness playing a partial mediating role. By applying causal inference methods that account for time-varying confounding, these findings highlight the potential significance of psychosocial pathways in this association. Population-level policies aimed at maintaining chewing function may also be beneficial for mental well-being in aging populations. Future research should examine whether interventions targeting chewing disability can help reduce loneliness and lower the risk of depressive symptoms in older adults.

## Supplementary Material

igaf100_Supplementary_Data

## Data Availability

The data underlying this article are available in the Centre for Ageing Research and Education, Duke-NUS Medical School. Restrictions apply to the availability of these data, which were used under license for this study. Data are available from the authors upon reasonable request, with the permission of Duke-NUS Medical School and other relevant stakeholders. This study was not preregistered.
