# Supplementary material for: Estimating the causal impact of chewing disability on depressive symptoms mediated by loneliness: a longitudinal marginal structural model study of older adults in Singapore
Source: Innov Aging. 2025 Sep 16;9(10):igaf100. doi: 10.1093/geroni/igaf100 (PMC12558686; doi:10.1093/geroni/igaf100)
Supplement: igaf100_Supplementary_Data [file igaf100_supplementary_data.docx]

***Innovation in Aging* Supplementary Material:** **Estimating the causal impact of chewing disability on depressive symptoms mediated by loneliness: Tay, Nascimento, Chan, Malhotra, Tonetti, & Peres.** **A longitudinal marginal structural model study of older adults in Singapore**

**Supplementary Table 1.** Chewing disability groupings.

**Supplementary Table 2.** Baseline and follow‐up characteristics of participants with complete data from Wave 1 (2009) through Wave 3 (2015), before multiple imputation.

**Supplementary Table 3.** Characteristics of eligible participants at each wave before multiple imputation, without accounting for loss to follow-up.

**Supplementary Table 4.** Distribution of missing data in the analytic sample addressed through multiple imputation.

**Supplementary Table 5.** Characteristics of participants from Wave 1 (2009) to Wave 3 (2015), comparing (A) the imputed dataset (baseline CSDS not excluded; CSDS and loneliness modelled as continuous scores), (B) the complete case sample prior to multiple imputation, and (C) the unimputed dataset before accounting for loss to follow-up.

**Supplementary Table 6.** Stabilized inverse probability weights diagnostics before and after weight truncation.

**Supplementary Table 7.** Estimated impact of alternative chewing disability classification (Group 1 & 2 = no chewing disability) on CSDS using inverse probability treatment weighting and marginal structural models.

**Supplementary Table 8.** Estimated impact of chewing disability on CSDS using alternative loneliness classification (Mostly lonely: Three-Item Loneliness Scale ≥4).

**Supplementary Table 9.** Estimated impact of chewing disability on CSDS, excluding participants with baseline cognitive impairment.

**Supplementary Table 10.** Sensitivity analysis for non-random attrition at wave 3 under best- and worst-case assumptions.

**Supplementary Table 11.** Estimated impact of chewing disability on CSDS, with both outcome (CSDS) and mediator (loneliness) modelled as continuous scores (CED-D and TILS).

**Supplementary Figure 1.** Density plot of stabilized inverse probability weights (IPW) before (orange) and after truncation (teal), based on imputed data.

**Supplementary Figure 2.** Distribution of predicted probabilities from regression models for the exposure and mediator to check for potential positivity violations at Waves 1 and 2.

**Supplementary Table 1.** Chewing disability groupings.

| Participants were asked, “The following foods are ordered from hardest to softest to chew. What is the hardest group you are able to bite and chew?” The response options were grouped based on food toughness contextualised to local foods commonly consumed by older adults in Singapore, and chewing muscle activity. This was grouped in descending order of toughness:  **Group 1**: Ikan Bilis (dried anchovies) in Nasi Lemak or shredded dry squid  **Group 2**: Mutton curry, dry mango, or fresh carrots,  **Group 3**: Bak-kwa, bread with crust not toasted, or kang kong steam boiled, chicken satay, or raw cucumber  **Group 4**: Thai rice, fried fish balls, or wanton noodles  **Group 5**: Bananas, ripe papaya, hard-boiled egg  **Group 6**: Unable to chew the foods listed in group 5 |
| --- |

**Supplementary Table 2.** Baseline and follow‐up characteristics of participants with complete data from Wave 1 (2009) through Wave 3 (2015), before multiple imputation.

|  | **Wave 1 (2009)** | **Wave 2 (2011-2012)** | | | **Wave 3 (2015)** | |
| --- | --- | --- | --- | --- | --- | --- |
|  | ***N* = 1,165** | **No CSDS  *n* = 1,101** | **With CSDS *n* = 64** | **No CSDS *n* = 1,043** | | **With CSDS *n* = 122** |
|  | Mean (SD)  or *N* (%) | Mean (SD)  or *N* (%) | Mean (SD)  or *N* (%) | Mean (SD)  or *N* (%) | | Mean (SD)  or *N* (%) |
| Age | 69.8 (6.7) | 71.7 (6.7) | 72.2 (7.2) | 75.7 (6.8) | | 76.0 (6.5) |
| Sex |  |  |  |  | |  |
| Male | 522 (44.8) | 506 (46.0) | 16 (25.0) | 473 (45.3) | | 49 (40.2) |
| Female | 643 (55.2) | 595 (54.0) | 48 (75.0) | 570 (54.7) | | 73 (59.8) |
| Ethnicity |  |  |  |  | |  |
| Chinese | 864 (74.2) | 815 (74.0) | 49 (76.6) | 775 (74.3) | | 89 (73.0) |
| Malay | 193 (16.6) | 187 (17.0) | 6 (9.4) | 172 (16.5) | | 21 (17.2) |
| Indian | 95 (8.2) | 86 (7.8) | 9 (14.1) | 85 (8.1) | | 10 (8.2) |
| Others | 13 (1.1) | 13 (1.2) | 0 (0.0) | 11 (1.1) | | 2 (1.6) |
| Education |  |  |  |  | |  |
| Primary school or below | 785 (67.4) | 738 (67.0) | 47 (73.4) | 693 (66.4) | | 92 (75.4) |
| Above primary school | 380 (32.6) | 363 (33.0) | 17 (26.6) | 350 (33.6) | | 30 (24.6) |
| Housing |  |  |  |  | |  |
| 1-2 room government-built | 80 (6.9) | 67 (6.1) | 13 (20.3) | 64 (6.1) | | 16 (13.1) |
| 3 room government-built | 328 (28.2) | 310 (28.2) | 18 (28.1) | 291 (27.9) | | 37 (30.3) |
| 4-5 room government-built/private housing | 757 (65.0) | 724 (65.8) | 33 (51.6) | 688 (66.0) | | 69 (56.6) |
| Smoking |  |  |  |  | |  |
| Non-/Ex-smoker | 1,045 (89.7) | 1,003 (91.1) | 60 (93.8) | 954 (91.5) | | 116 (95.1) |
| Current | 120 (10.3) | 98 (8.9) | 4 (6.3) | 89 (8.5) | | 6 (4.9) |
| Cerebrovascular disease |  |  |  |  | |  |
| Yes | 22 (1.9) | 19 (1.7) | 4 (6.3) | 32 (3.1) | | 11 (9.0) |
| No | 1,143 (98.1) | 1,082 (98.3) | 60 (93.8) | 1,011 (96.9) | | 111 (91.0) |
| Coronary heart disease |  |  |  |  | |  |
| Yes | 60 (5.2) | 45 (4.1) | 5 (7.8) | 78 (7.5) | | 17 (13.9) |
| No | 1,105 (94.8) | 1,056 (95.9) | 59 (92.2) | 965 (92.5) | | 105 (86.1) |
| Diabetes mellitus |  |  |  |  | |  |
| Yes | 264 (22.7) | 257 (23.3) | 23 (35.9) | 309 (29.6) | | 47 (38.5) |
| No | 901 (77.3) | 844 (76.7) | 41 (64.1) | 734 (70.4) | | 75 (61.5) |
| Cancer |  |  |  |  | |  |
| Yes | 23 (2.0) | 10 (0.9) | 2 (3.1) | 46 (4.4) | | 4 (3.3) |
| No | 1,142 (98.0) | 1,091 (99.1) | 62 (96.9) | 997 (95.6) | | 118 (96.7) |
| Mobility difficulty |  |  |  |  | |  |
| Yes | 324 (27.8) | 326 (29.6) | 31 (48.4) | 515 (49.4) | | 86 (70.5) |
| No | 841 (72.2) | 775 (70.4) | 33 (51.6) | 528 (50.6) | | 36 (29.5) |
| Social support network (modified LSNS-R) | 30.4 (12.4) | 29.5 (10.6) | 23.9 (9.4) | 27.3 (11.4) | | 24.2 (10.4) |
| Chewing disability |  |  |  |  | |  |
| Yes | 188 (16.1) | 173 (15.7) | 21 (32.8) | 245 (23.5) | | 27 (22.1) |
| No | 977 (83.9) | 928 (84.3) | 43 (67.2) | 798 (76.5) | | 95 (77.9) |
| Loneliness (TILS ≥1) |  |  |  |  | |  |
| Yes | 465 (39.9) | 367 (33.3) | 48 (75.0) | 388 (37.2) | | 90 (73.8) |
| No | 700 (60.1) | 734 (66.7) | 16 (25.0) | 655 (62.8) | | 32 (26.2) |

*Notes:* CSDS, clinically significant depressive symptoms; LSNS-R, Lubben Social Network Scale-Revised; N, number; SD, standard deviation; TILS, Three-Item Loneliness Scale.

**Supplementary Table 3.** Characteristics of eligible participants at each wave before multiple imputation, without accounting for loss to follow-up.

|  | **Wave 1 (2009)** | **Wave 2 (2011-2012)** | | **Wave 3 (2015)** | |
| --- | --- | --- | --- | --- | --- |
|  | ***N* = 3,797** | **No CSDS  *n* = 2,156** | **With CSDS  *n* = 152** | **No CSDS  *n* = 1,043** | **With CSDS  *n* = 122** |
|  | Mean (SD)  or *N* (%) | Mean (SD)  or *N* (%) | Mean (SD)  or *N* (%) | Mean (SD)  or *N* (%) | Mean (SD)  or *N* (%) |
| Age | 71.7 (7.6) | 72.8 (7.1) | 74.3 (7.8) | 75.7 (6.8) | 76.0 (6.5) |
| Sex |  |  |  |  |  |
| Male | 1,837 (48.4) | 1,074 (49.8) | 62 (40.8) | 473 (45.3) | 49 (40.2) |
| Female | 1,960 (51.6) | 1,082 (50.2) | 90 (59.2) | 570 (54.7) | 73 (59.8) |
| Ethnicity |  |  |  |  |  |
| Chinese | 2,741 (72.2) | 1,585 (73.5) | 104 (68.4) | 775 (74.3) | 89 (73.0) |
| Malay | 632 (16.6) | 348 (16.1) | 27 (17.8) | 172 (16.5) | 21 (17.2) |
| Indian | 379 (10.0) | 198 (9.2) | 21 (13.8) | 85 (8.1) | 10 (8.2) |
| Others | 45 (1.2) | 25 (1.2) | 0 (0.0) | 11 (1.1) | 2 (1.6) |
| Education |  |  |  |  |  |
| Primary school or below | 2,643 (69.6) | 1,439 (66.7) | 108 (71.1) | 693 (66.4) | 92 (75.4) |
| Above primary school | 1,154 (30.4) | 717 (33.3) | 44 (28.9) | 350 (33.6) | 30 (24.6) |
| Housing |  |  |  |  |  |
| 1-2 room government-built | 298 (7.8) | 167 (7.7) | 27 (17.8) | 64 (6.1) | 16 (13.1) |
| 3 room government-built | 997 (26.3) | 560 (26.0) | 44 (28.9) | 291 (27.9) | 37 (30.3) |
| 4-5 room government-built/private housing | 2,502 (65.9) | 1,429 (66.3) | 81 (53.3) | 688 (66.0) | 69 (56.6) |
| Smoking |  |  |  |  |  |
| Non-/Ex-smoker | 3,372 (88.8) | 1,946 (90.3) | 137 (90.1) | 954 (91.5) | 116 (95.1) |
| Current | 425 (11.2) | 210 (9.7) | 15 (9.9) | 89 (8.5) | 6 (4.9) |
| Cerebrovascular disease |  |  |  |  |  |
| Yes | 112 (2.9) | 45 (2.1) | 9 (5.9) | 16 (1.5) | 6 (4.9) |
| No | 3,685 (97.1) | 2,111 (97.9) | 143 (94.1) | 1,027 (98.5) | 116 (95.1) |
| Coronary heart disease |  |  |  |  |  |
| Yes | 245 (6.5) | 102 (4.7) | 12 (7.9) | 78 (7.5) | 17 (13.9) |
| No | 3,552 (93.5) | 2,054 (95.3) | 140 (92.1) | 965 (92.5) | 105 (86.0) |
| Diabetes mellitus |  |  |  |  |  |
| Yes | 864 (22.8) | 493 (22.9) | 50 (32.9) | 309 (29.6) | 47 (38.5) |
| No | 2,933 (77.2) | 1,663 (77.1) | 102 (67.1) | 734 (70.4) | 75 (61.5) |
| Cancer |  |  |  |  |  |
| Yes | 103 (2.7) | 31 (1.4) | 5 (3.3) | 20 (1.9) | 3 (2.5) |
| No | 3,694 (97.3) | 2,125 (98.6) | 147 (96.7) | 1,023 (98.1) | 119 (97.5) |
| Mobility difficulty |  |  |  |  |  |
| Yes | 1,218 (32.1) | 695 (32.2) | 83 (54.6) | 270 (25.9) | 54 (44.3) |
| No | 2,579 (67.9) | 1,461 (67.8) | 69 (45.4) | 773 (74.1) | 68 (55.7) |
| Social support network (modified LSNS-R) | 29.5 (12.8) | 28.6 (10.6) | 23.5 (9.9) | 27.3 (11.4) | 24.2 (10.4) |
| Chewing disability |  |  |  |  |  |
| Yes | 752 (19.8) | 618 (28.7) | 80 (52.6) | 153 (14.7) | 35 (28.7) |
| No | 3,045 (80.2) | 1,538 (71.3) | 72 (47.4) | 890 (85.3) | 87 (71.3) |
| Loneliness (TILS ≥1) |  |  |  |  |  |
| Yes | 1,700 (44.8) | 786 (36.5) | 105 (69.1) | 410 (39.3) | 55 (45.1) |
| No | 2,097 (55.2) | 1,370 (63.5) | 47 (30.9) | 633 (60.7) | 67 (54.9) |

*Notes:* CSDS, clinically significant depressive symptoms; LSNS-R, Lubben Social Network Scale-Revised; N, number; SD, standard deviation; TILS, Three-Item Loneliness Scale.

**Supplementary Table 4.** Distribution of missing data in the analytic sample addressed through multiple imputation.

| Covariate | Number missing | Proportion missing |
| --- | --- | --- |
| Education | 1 | 0.1 |
| Social support network score (Wave 2) | 40 | 3.1 |
| Social support network score (Wave 3) | 94 | 7.4 |
| Loneliness score (Wave 2) | 45 | 3.5 |
| Loneliness score (Wave 3) | 94 | 7.4 |

**Supplementary Table 5.** Characteristics of participants from Wave 1 (2009) to Wave 3 (2015), comparing (A) the imputed dataset (baseline CSDS not excluded; CSDS and loneliness modelled as continuous scores), (B) the complete case sample prior to multiple imputation, and (C) the unimputed dataset before accounting for loss to follow-up.

1. Imputed dataset (N = 1,517)

|  | **Wave 1 (2009)** | **Wave 2 (2011-2012)** | **Wave 3 (2015)** |
| --- | --- | --- | --- |
|  | Mean (SD) or Column, % | Mean (SD) or Column, % | Mean (SD) or Column, % |
| Chewing disability |  |  |  |
| Yes | 21.3 | 29.0 | 35.8 |
| No | 78.7 | 71.0 | 64.2 |
| CES-D | 3.4 (3.1) | 2.5 (2.7) | 3.2 (3.2) |
| TILS | 1.8 (2.3) | 1.3 (2.0) | 2.2 (3.3) |

1. Complete case sample (N = 1,377)

|  | **Wave 1 (2009)** | **Wave 2 (2011-2012)** | **Wave 3 (2015)** |
| --- | --- | --- | --- |
|  | Mean (SD) or Column, % | Mean (SD) or Column, % | Mean (SD) or Column, % |
| Chewing disability |  |  |  |
| Yes | 255 (18.5%) | 304 (22.1%) | 509 (37.0%) |
| No | 1,122 (81.5%) | 1,073 (77.9%) | 868 (63.0%) |
| CES-D | 3.3 (3.0) | 2.4 (2.6) | 3.1 (3.1) |
| TILS | 1.7 (2.3) | 1.3 (1.9) | 2.2 (3.4) |

1. Unimputed dataset (N = 4,530 at Wave 1; N = 2,726 at Wave 2; N = 1,377 at Wave 3)

|  | **Wave 1 (2009)** | **Wave 2 (2011-2012)** | **Wave 3 (2015)** |
| --- | --- | --- | --- |
|  | Mean (SD) or Column, % | Mean (SD) or Column, % | Mean (SD) or Column, % |
| Chewing disability |  |  |  |
| Yes | 1,030 (22.7%) | 852 (31.3%) | 509 (37.0%) |
| No | 3,500 (77.3%) | 1,874 (68.7%) | 868 (63.0%) |
| CES-D | 3.5 (3.2) | 2.6 (2.7) | 3.1 (3.1) |
| TILS | 1.0 (0.0, 3.0) | 1.3 (1.9) | 2.3 (3.4) |

*Notes:* CES-D, Center for Epidemiologic Studies – Depression; CSDS, clinically significant depressive symptoms; N, number; SD, standard deviation; TILS, Three-Item Loneliness Scale.

**Supplementary Table 6.** Stabilized inverse probability weights diagnostics before and after weight truncation.

| Statistic | Imputed data | | Complete case analysis | |
| --- | --- | --- | --- | --- |
|  | No truncation | 95% truncation | No truncation | 95% truncation |
| Mean | 1.43 | 1.31 | 1.16 | 1.08 |
| Standard deviation | 1.41 | 0.85 | 0.82 | 0.48 |
| Minimum | 0.10 | 0.10 | 0.22 | 0.22 |
| 1^st^ percentile | 0.19 | 0.19 | 0.38 | 0.38 |
| 95^th^ percentile | 7.67 | 3.67 | 2.43 | 2.19 |
| 99^th^ percentile | 7.67 | 3.67 | 4.97 | 2.19 |
| Maximum | 21.19 | 3.67 | 10.29 | 2.19 |

**Supplementary Table 7.** Estimated impact of alternative chewing disability classification (Group 1 & 2 = no chewing disability) on CSDS using inverse probability treatment weighting and marginal structural models.

| Variable | Imputed data (*n* = 1,277) | | | | Complete case analysis (*n* = 1,165) | | | |
| --- | --- | --- | --- | --- | --- | --- | --- | --- |
|  | **RR** | **95% CI** | **E-value** | **Proportion contributed** | **RR** | **95% CI** | **E-value** | **Proportion contributed** |
| Total cumulative effect (Model 1) | 1.57 | 1.24-1.91 | 2.53 | 100% | 1.70 | 1.32-2.14 | 2.78 | 100% |
| Direct cumulative effect (Model 2) | 1.50 | 1.18-1.83 | 2.37 | 87.50% | 1.65 | 1.28-2.09 | 2.70 | 94.09% |
| Cumulative mediator effect (loneliness) | 1.22 | 0.95-1.48 | 1.73 | 12.50% | 1.20 | 0.94-1.56 | 1.70 | 5.91% |

*Notes:* CI, confidence interval; RR, relative risk. Models adjusted for age, sex, ethnicity, education, housing type, cerebrovascular disease, coronary heart disease, diabetes mellitus, cancer, social support network. Model 2 additionally adjusts for loneliness as a mediator. CIs for the complete-case analysis were obtained using nonparametric bootstrap with 5,000 replicates.

**Supplementary Table 8.** Estimated impact of chewing disability on CSDS using alternative loneliness classification (Mostly lonely: Three-Item Loneliness Scale ≥4).

| Variable | Imputed data (*n* = 1,277) | | | | Complete case analysis (*n* = 1,165) | | | | |  |
| --- | --- | --- | --- | --- | --- | --- | --- | --- | --- | --- |
|  | RR | 95% CI | E-value | Proportion contributed | | RR | 95% CI | E-value | Proportion contributed | |
| Total cumulative effect (Model 1) | 1.44 | 1.12-1.77 | 2.24 | 100% | | 1.35 | 1.06-1.74 | 2.05 | 100% | |
| Direct cumulative effect (Model 2) | 1.35 | 1.02-1.67 | 2.03 | 78.21% | | 1.33 | 1.03-1.71 | 1.99 | 92.29% | |
| Cumulative mediator effect (loneliness) | 1.70 | 1.30-2.09 | 2.78 | 21.79% | | 1.58 | 1.12-2.15 | 2.54 | 7.71% | |

*Notes:* CI, confidence interval; RR, relative risk. Models adjusted for age, sex, ethnicity, education, housing type, cerebrovascular disease, coronary heart disease, diabetes mellitus, cancer, social support network. Model 2 additionally adjusts for loneliness as a mediator. CIs for the complete-case analysis were obtained using nonparametric bootstrap with 5,000 replicates.

**Supplementary Table 9.** Estimated impact of chewing disability on CSDS, excluding participants with baseline cognitive impairment.

| Variable | Imputed data (*n* = 1,072) | | | | Complete case analysis (*n* = 1,008) | | | |
| --- | --- | --- | --- | --- | --- | --- | --- | --- |
|  | RR | 95% CI | E-value | Proportion contributed | RR | 95% CI | E-value | Proportion contributed |
| Total cumulative effect (Model 1) | 1.51 | 1.20-1.83 | 2.39 | 100% | 1.32 | 0.99-1.76 | 1.98 | 100% |
| Direct cumulative effect (Model 2) | 1.42 | 1.10-1.75 | 2.20 | 82.44% | 1.30 | 0.96-1.74 | 1.93 | 93.72% |
| Cumulative mediator effect (loneliness) | 1.27 | 0.96-1.57 | 1.85 | 17.56% | 1.17 | 0.88-1.57 | 1.62 | 6.28% |

*Notes:* CI, confidence interval; RR, relative risk. Models adjusted for age, sex, ethnicity, education, housing type, cerebrovascular disease, coronary heart disease, diabetes mellitus, cancer, social support network. Model 2 additionally adjusts for loneliness as a mediator. CIs for the complete-case analysis were obtained using nonparametric bootstrap with 5,000 replicates.

**Supplementary Table 10.** Sensitivity analysis for non-random attrition at wave 3 under best- and worst-case assumptions.

| Variable | Best-case scenario | Worst-case scenario |
| --- | --- | --- |
|  | RR (95% CI) | |
| Total cumulative effect (Model 1) | 1.53 (1.19–1.97) | 1.13 (1.05–1.22) |
| Direct cumulative effect (Model 2) | 1.44 (1.13–1.83) | 1.13 (1.05–1.22) |
| Cumulative mediator effect (loneliness) | 1.17 (0.92–1.49) | 1.06 (0.98–1.14) |

*Notes:* CI, confidence interval; RR, relative risk.

**Supplementary Table 11.** Estimated impact of chewing disability on CSDS, with both outcome (CSDS) and mediator (loneliness) modelled as continuous scores (CED-D and TILS).

| Variable | Imputed data (*n* = 1,517) | | | | Complete case analysis (*n* = 1,377) | | | |
| --- | --- | --- | --- | --- | --- | --- | --- | --- |
|  | MD  (95% CI) | Approx. RR  (95% CI) | E-value | Proportion contributed | MD  (95% CI) | Approx. RR  (95% CI) | E-value | Proportion contributed |
| Total cumulative effect (Model 1) | 0.90 (0.61-1.19) | 1.29 (1.19-1.40) | 1.90 | 100% | 0.55 (0.25-0.88) | 1.17 (1.07-1.29) | 1.62 | 100% |
| Direct cumulative effect (Model 2) | 0.66 (0.33-1.00) | 1.20 (1.10-1.32) | 1.27 | 73.53% | 0.45 (0.16-0.78) | 1.14 (1.04-1.25) | 1.54 | 81.40% |
| Cumulative mediator effect (loneliness) | 0.17 (0.09-0.24) | 1.05 (1.03-1.07) | 1.70 | 26.47% | 0.16 (0.09-0.22) | 1.05 (1.03-1.07) | 1.27 | 18.60% |

*Notes:* Results are presented as mean differences, with approximate risk ratios derived. The proportion mediated was calculated on the mean difference scale.

Approx, approximate; CES-D, Center for Epidemiologic Studies – Depression; CI, confidence interval; MD, mean difference; RR, risk ratio; TILS, Three-Item Loneliness Scale. Models adjusted for age, sex, ethnicity, education, housing type, cerebrovascular disease, coronary heart disease, diabetes mellitus, cancer, social support network. Model 2 additionally adjusts for loneliness as a mediator. CIs for the complete-case analysis were obtained using nonparametric bootstrap with 5,000 replicates.

**Supplementary Figure 1.** Density plot of stabilized inverse probability weights (IPW) before (orange) and after truncation (teal), based on imputed data.


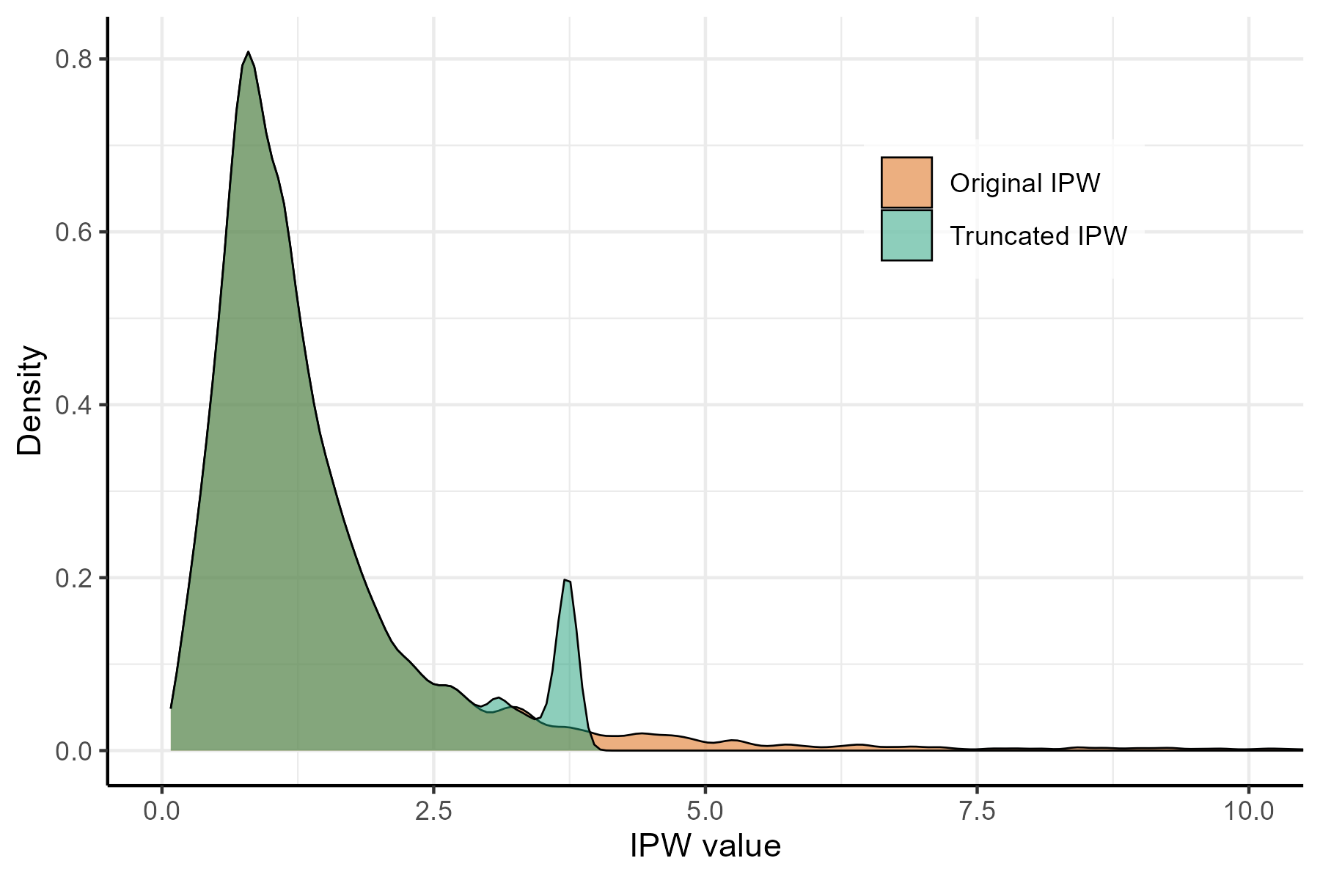


*Notes:* The darker green area shows where the two distributions overlap, illustrating how truncation removes extreme weights and reduces variability.

**Supplementary Figure 2.** Distribution of predicted probabilities from regression models for the exposure and mediator to check for potential positivity violations at Waves 1 and 2.


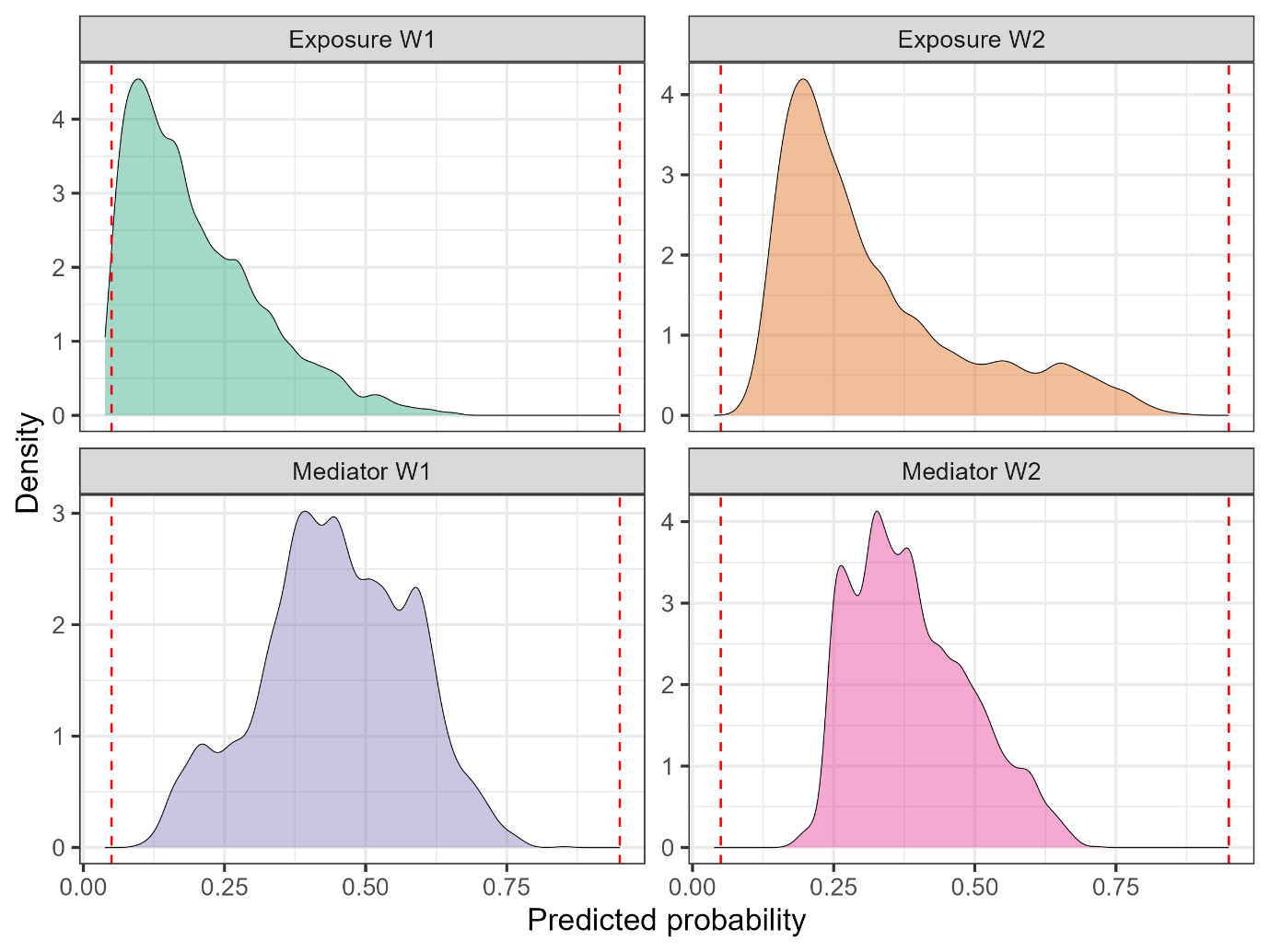


*Notes:* Red dashed lines mark the 5% and 95% thresholds. Each panel shows the distribution of predicted probabilities for the exposure or mediator model at the respective wave. Only the exposure model at Wave 1 shows a small proportion (1.98%) of predicted values below 0.05, suggesting limited potential for non-positivity. All other models show distributions within the acceptable central range.
